# Obituary.—Dr. Henry W. Dean

**Published:** 1878-02

**Authors:** 


DIED at Rochester, N. Y., January 13th, 1878. Dr. HENRY W. DEAN, in the 59th year of his age.

Dr. Dean was born in Madison Co., N. Y., in August 1818. His father removed, while he was yet a lad, to Wyoming Co. His academic studies were pursued at the Wyoming Academy. At the termination of this course, he commenced the study of medicine with Dr. W. Fay of Pavilion, N. Y. He came to Rochester, N. Y., in 1839 and entered the office of Dr. Frank H. Hamilton, as a student. He graduated at the Geneva Medical College in 1842, since which time he has continued the practice of his profession in Rochester.

His illness was brief, he having attended to his duties, as usual, until two days before his death, which occurred from Meningeal Apoplexy.

Few Medical Men, in Western New York, have enjoyed the confidence of the profession and public to the same extent as Dr. Dean. Of commanding presence, calm, dignified and sympathetic, he impressed every one, with whom he came in contact, with the magnetism of blended strength and sweetness. In his professional labors, his patient and constant study made him the judicious counsellor and physician. In all his relations, his careful consideration of others won for him the affection of all who knew him. While even the student of science, an earnest religious zeal was the motive impulse of his life. He held "the mystery of the faith in a pure conscience." For him his calling involved a sacred duty; that of reflection, in his work and life, the character of the christian physician. In his relations with his professional brethren, his uniform courtesy and urbanity won their profound esteem and regard. His attainments, though modestly displayed were recognized and gained for him the places of honor and distinction in the profession. To the elevation of the calling, which he so truly loved, he was a devoted laborer; and his earnest effort was, ever, to free it from all that seemed base or ignoble.

That his life should terminate suddenly, and without a struggle, seems very fitting. It was but the sudden ending of a strain of harmony, and he quietly passed from this life

> "As one who wraps the drapery of his couch about him
> And lies down to pleasant dreams."

E. V. S.

At a meeting of the Monroe County Medical Society, held January 15th, the following Resolutions were unanimously adopted :

Resolved, That in the death of Dr. Henry W. Dean this society has lost one of its oldest, most able and efficient members—one always ready and foremost in every work for the promotion of the scientific interests of the profession.

Resolved, That as friends and associates of Dr. Dean, we can bear unqualified testimony to his great personal worth, and to the kindness and liberality which characterised all his professional relations

Resolved, That we share with this community in regrets for the loss of an eminently useful man and honored physician, and that we tender our sympathy, with these expressions of regard for our deceased friend, to his bereaved family.

